# Reflected Wave Reduction Based on Time-Delay Separation for the Plane Array of Multilayer Acoustic Absorbers

**DOI:** 10.3390/s21248432

**Published:** 2021-12-17

**Authors:** Hwijin Park, Yeong Bae Won, Sehyeong Jeong, Joo Young Pyun, Kwan Kyu Park, Jeong-Min Lee, Hee-Seon Seo, Hak Yi

**Affiliations:** 1 Department of Mechanical Engineering, Kyungpook National University, Daegu 41566, Korea; phj0917@knu.ac.kr (H.P.); wyp1028s@knu.ac.kr (Y.B.W.); jsh9634@knu.ac.kr (S.J.); 2 Department of Convergence Mechanical Engineering, Hanyang University, Seoul 04763, Korea; jooyoungpyun@hanyang.ac.kr (J.Y.P.); kwankyu@hanyang.ac.kr (K.K.P.); 3 Maritime Technology Research Institute, Agency for Defense Development, Changwon 51682, Korea; leemin@add.re.kr (J.-M.L.); hsseo@add.re.kr (H.-S.S.)

**Keywords:** smart skin, acoustic absorber, active noise control, piezoelectric material

## Abstract

This paper presents a control technique for reducing the reflection of acoustic signals for the plane array of multilayer acoustic absorbers underwater. In order to achieve this, a plane array of multilayer acoustic absorbers is proposed to attenuate low-frequency noise, with each unit consisting of a piezoelectric transducer, two layers of polyvinylidene fluorides and three layers of the acoustic window. Time-delay separation is used to find the incident and reflected acoustic signals to achieve reflected sound reduction. Experimental comparison of the attenuation rate of the reflected acoustic signal when performing passive and active controls is considered to verify the effectiveness of the time-delay separation technique applied plane array absorbers. Experiments on the plane array of smart skin absorbers confirmed that the reduction of reflected acoustic signals makes it suitable for a wide range of underwater applications.

## 1. Introduction

A passive acoustic absorber has traditionally been used as a method of acoustic absorption underwater. Because of the material properties of passive acoustic absorbers, the passive sound-absorbing strategy is dependent on the thickness of the absorber to block the path of the reflection signal [1,2,3,4]. To overcome this limit, active noise control (ANC), a combination of smart material and an active digital controller, was researched.

To create a quiet space, ANC generates a suitable controlled acoustic signal with the same magnitude but opposite phase against the incident wave [5,6,7]. It enables the physical size of the passive acoustic absorber to be smaller and lighter in the low-frequency acoustic absorption when compared to the passive one. Furthermore, digital controllers can provide high accuracy with adjustable performance and flexibility [8,9,10,11].

Meanwhile, to reduce the reflected acoustic signal, it is critical to separate the incident and reflected waves that exist in the same path. In order to accomplish this, time-delay signal separation is used to analyze superposed signals and separate them into multiple independent signals [12,13,14]. This technique’s implementation is based on precise measurements and is less dependent on the dynamic model of sensors. At least two receiving sensors are required to apply time-delay signal separation [13,14]. These interesting researches are also used for sensor arrays to measure a propagating wave field [15] and the time difference of arrival estimation [16,17].

Many efforts are being made to convert a single acoustic sensor into a plane array to achieve meaningful absorption underwater performance [6,18,19,20]. However, there was still not a plane array with a multilayer acoustic absorber for attenuating reflected waves. A control scheme to attenuate the reflected acoustic wave for multilayer acoustic absorbers was still investigated [18,19].

This paper is organized as follows. Section 2 describes the proposed multilayer absorber system, and Section 3 discusses the transmitting and receiving sensitivities of the acoustic absorber system. Section 4 describes a noise cancellation strategy for the multilayer acoustic absorber system. Section 5 discusses the findings of echo reduction experiments carried out in this study. Finally, Section 6 summarizes the findings.

## 2. Design of Multilayer Acoustic Absorber

### 2.1. Single Multilayer Acoustic Absorbers

**Figure 1:**
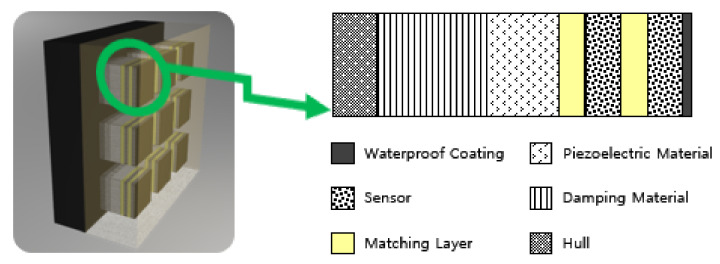
Concept of the smart skin system.

**Figure 2:**
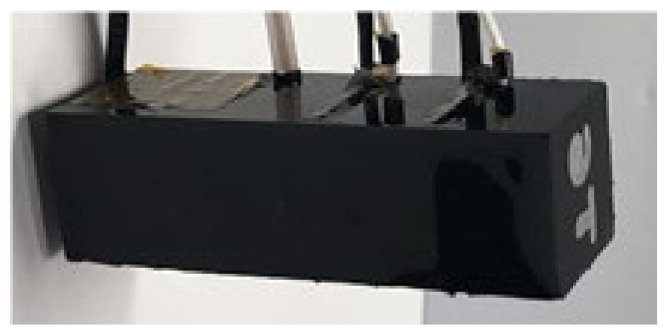
Single smart skin absorber.

Figure 1 shows the proposed single, smart skin absorber in this study, which is stacked with a PZT-5A as an acoustic transducer, two layers of polyvinylidene fluorides (PVDFs) as a receiving sensor and three layers of Rho-c rubber as acoustic windows [14]. Its size is a total of 18 mm × 18 mm × 60.12 mm. The appearance of the proposed system that is finally fabricated is shown in Figure 2.

In the developed absorber, a piezoelectric transducer with a high transmitting sensitivity serves to reduce the reflected acoustic signal that originates from the surface of the piezoelectric material. In order to complete this, the piezoelectric transducer responds to the incident signal by simultaneously sending a controlled acoustic signal with the opposite phase. Two receiving sensors are placed in quarter wavelength and half wavelength, respectively, along the thickness direction from the piezoelectric transducer. The goal of the receiving sensors in the single, smart skin absorber is to collect acoustic signals superposed on the incident, reflected and control signals. Finally, Rho-c acoustic windows with a low reflected value are used to match the acoustic impedance of the window to the impedance of the water. The total system is fabricated in a stacked form, which is covered by the Kapton tape.

### 2.2. Plane Array of Multilayer Acoustic Absorbers

**Figure 3:**
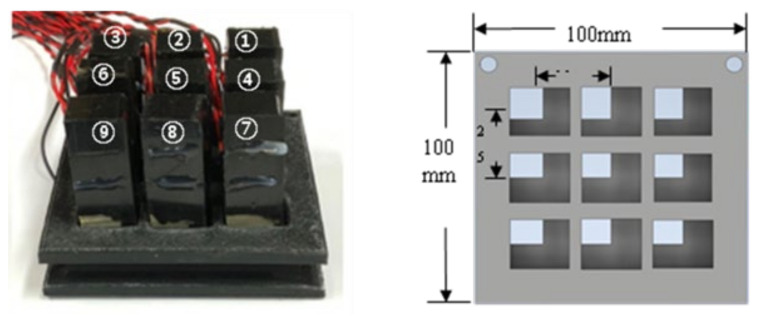
Plane array of smart skin sensors and designed tray.

In this study, the plane array of smart skin absorbers is realized by installing each absorber on a tray (mold) of 3D printed material. The proposed plane array has a size of 68 mm × 68 mm × 60.12 mm and is made up of nine single, smart skin absorbers arranged in three rows and three columns. Figure 3 depicts the appearance of the proposed system and designed tray that was eventually fabricated. In order to avoid the complexities of multisensor electric cables, all signal lines in the electrodes are routed through the bottom space on a tray in the developed absorber. There is a total of 18 receiving channels and nine control output channels.

## 3. Acoustic Property of the Multilayer Acoustic Absorbers in Single and Plane Array

To find the performance of high acoustic absorption, transmitting and receiving sensitivities in both single absorber and plane array are achieved. Its transmitting and receiving sensitivities were measured experimentally. These are determined by the properties of each material, such as acoustic impedance. The crosstalk voltage level between each single, smart skin absorber in a plane array is also analyzed.

Experimental measurement is conducted in a water tank (1 m × 0.75 m × 0.7 m). Both a projector for receiving sensitivity and a hydrophone (Teledyne TC4013) for transmitted sensitivity is positioned at 150 mm from the front of the developed plane array of smart skin absorbers, considering the far-field. A piezoelectric transducer in a single, smart skin absorber generates a targeting wave to the hydrophone during catch-up signal experiments on transmitted sensitivity. Two layers of PVDFs collect the incident acoustic signal generated by the projector in catch-up experiments on receiving sensitivity. In two cases, the transmitting and receiving sensitivities of the proposed multilayer acoustic absorbers are collected using STM32H743ZI as the main controller unit and AD5685R as the digital–analog converter (DAC): the single absorber and the plane array.

### 3.1. Property of Single Multilayer Acoustic Absorber

The transmitting sensitivity *T*(*ω*) of the piezoelectric transducer in the proposed skin can be defined as

(1) $$T\left(\omega \right)=\frac{V_{Output}}{V_{Input}\times S_{Tr}\left(\omega \right)}$$

$V_{Output}$ and $V_{Input}$ are the measured voltage and drive control signal of the piezoelectric composite transducer, respectively. $S_{Tr}\left(\omega \right)$ is the receiving sensitivity of the hydrophone.

**Figure 4:**
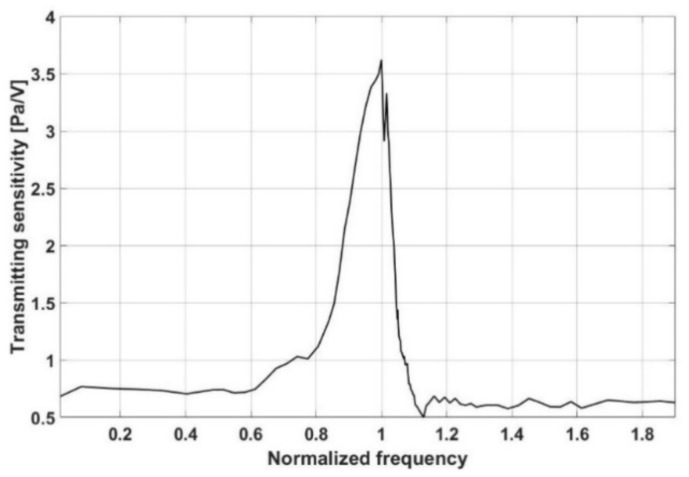
Transmitting sensitivity of the proposed single absorber.

Figure 4 shows the proposed absorber’s normalized transmitting sensitivity as determined by an experiment and plotted against a frequency range. Figure 4 depicts a significant decrease in transmitting voltage response around the target frequency. It implies that having a resonant frequency at the target frequency results in the best transmission efficiency.

**Figure 5:**
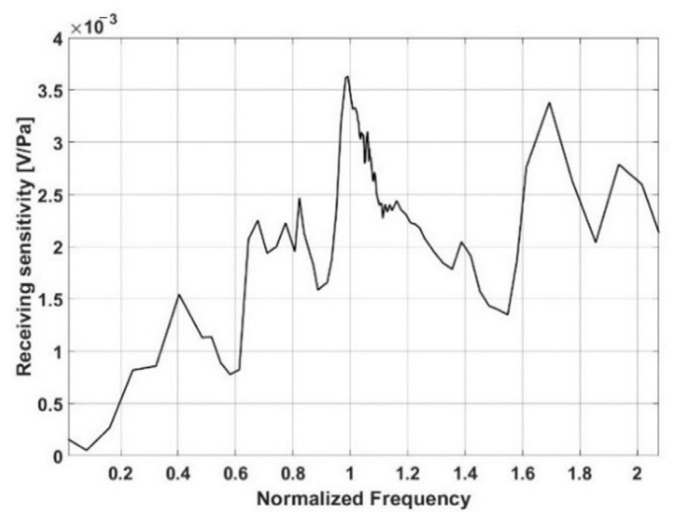
Receiving sensitivity of the proposed single, smart skin absorber.

Figure 5 shows the receiving sensitivity of the proposed single multilayer acoustic absorber, $S\left(\omega \right)$, and is calculated using the amplitude of the acoustic signal measured by the PVDFs.

(2) $$S\left(\omega \right)=\frac{V_{Output}\times d}{V_{Input}\times T_{Tr}\left(\omega \right)}$$

$V_{Output}$ and $V_{Input}$ are the voltage signal received by the PVDF and source signal of the projector, $T_{Tr}\left(\omega \right)$ is the transmitting sensitivity of the projector, respectively, and d is the distance between the sensor and projector.

Despite the presence of multiple peaks across a frequency range, the receiving sensitivity for a single, smart skin absorber shows that it is dominant around the targeting frequency, as shown in Figure 5. The plane array’s receiving sensors are all made up of the same absorber. Assume that a single absorber’s receiving property is the same as that of a plane array.

### 3.2. Property of a Plane Array with the Multilayer Acoustic Absorber

**Figure 6:**
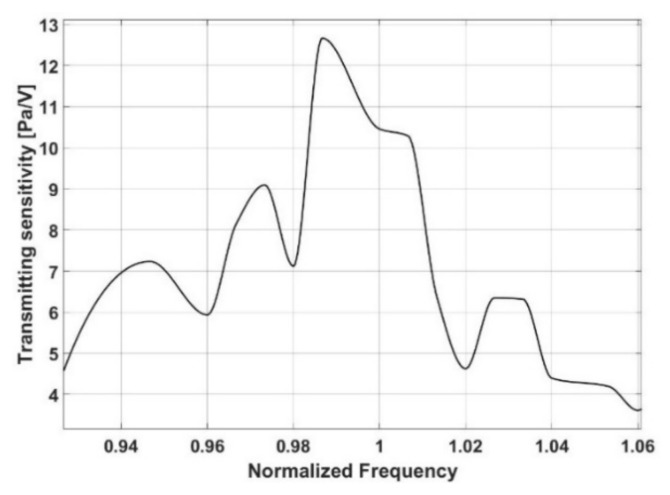
Transmit sensitivity of the proposed plane array.

Figure 6 shows the transmitting sensitivity of the proposed plane array with a multilayer acoustic absorber over a range of low frequencies. In particular, the transmitting sensitivities for a plane array show that it is dominant around the targeting frequency and rapidly decreases when away from the resonant frequency.

### 3.3. Crosstalk Voltage Level

The effect of mutual impedance is measured by looking at the crosstalk voltage level on the developed plane array. The crosstalk voltage level indicates the extent to which sound sources generated by other single transducers in the form of an array sensor have an effect. If the crosstalk voltage level is high, it indicates that interference with the acoustic signal coming from the outside sound source is occurring, so this is a factor that should be considered in the plane array.

(3) $$\mathrm{Crosstalk}\;\mathrm{voltage}\;\mathrm{level}=20\;\log_{10}(V_{0}/V_{pp})$$

Equation (3) expresses how to calculate the crosstalk voltage level. $V_{pp}$ represents the voltage applied to the piezoelectric transducer of a single, smart absorber. $V_{0}$ is measured voltage by another single absorber PVDF.

**Figure 7:**
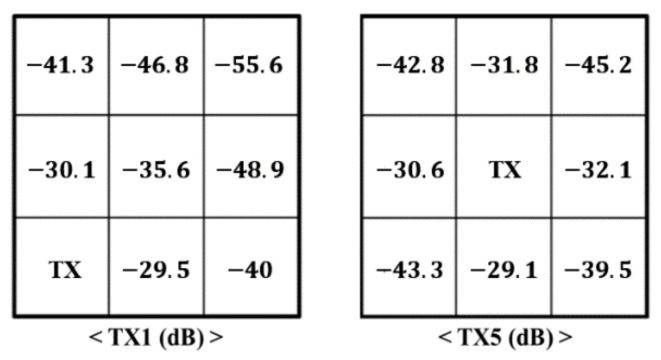
Crosstalk voltage level on TX1 and TX5.

As shown in Figure 7, the crosstalk voltage level of all single absorbers in the plane array of smart skin absorbers is less than −30 dB, implying that their mutual impedance is very low.

## 4. Reflection Reduction Control

### 4.1. Time-Delay Signal Separation

The incident wave travels perpendicular to the front of the proposed absorber, while the reflected wave travels in the opposite direction. This research uses the time-delay signal separation method in the use of two receiving sensors to separate the superposed acoustic signal. Measurement values of each receiving sensor, $V_{A}$, $V_{B}$, are as follows [14]:

(4) $$V_{A}=S\left(\omega \right)\left[P^{+}e^{2jkd}+P^{-}e^{-2jkd}\right]$$

(5) $$V_{B}=S\left(\omega \right)\left[P^{+}e^{jkd}+P^{-}e^{-jkd}\right]$$

$S\left(w\right)$ is the receiving sensitivity of the sensors, k is the wave number, d is the distance of the acoustic window between sensors and $P^{+}$ and $P^{-}$ are each amplitude of the incident and reflected acoustic signals, respectively. In order to compensate for the phase delay on each receiving sensor, the incident acoustic signal ($V_{in}$) to a sensor and reflected acoustic signal ($V_{r}$) from the front of the piezoelectric transducer are calculated as follows.

(6) $$V_{in}=V_{B}e^{-jkd}-V_{A}=-2jS\left(\omega \right)P^{+}\sin\left(kd\right)e^{jkd}$$

(7) $$e^{-jkd}-V_{r}=V_{A}V_{B}=-2jS\left(\omega \right)P^{-}\sin\left(kd\right)e^{-2jkd}$$

**Figure 8:**
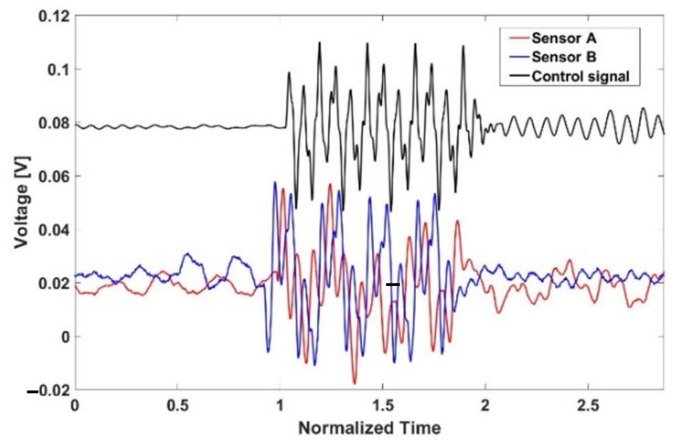
Measurement on two receiving sensors and the control signal.

Figure 8 displays the receiving data on each layer of PVDF and control signal. It is confirmed that there is a phase shift between receiving sensors.

### 4.2. Single Absorber

**Figure 9:**
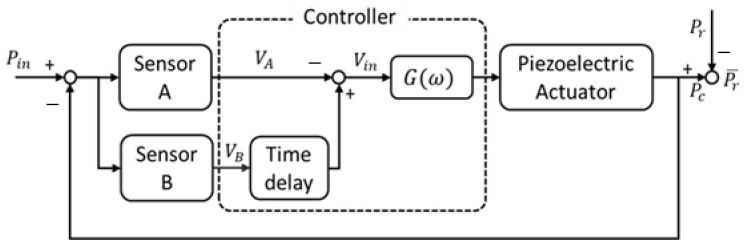
Block diagram of the proposed reduction control.

Figure 9 is a block diagram for controlling the reflected acoustic signal of a single, smart skin absorber. In the block diagram, the signal measured from the two receiving sensors of a single, smart skin absorber is divided into incidence acoustic signal and reflected acoustic signal through time-delay signal separation in the digital controller and multiplied by a constant gain (system gain) to drive the piezoelectric transducer.

The control output is generated to reduce the reflected acoustic signal from the front of the piezoelectric transducer using the concepts of active noise control and time-delay separation. The estimated reflected acoustic signal is calculated at the moment of impact with the front of the piezoelectric transducer by multiplying a reflection constant by the incident acoustic signal under the assumption of no energy loss.

Control wave is defined as a signal with the property of the equal amplitude and opposite phase of the estimated reflected wave; to match the control signal to reflected signals,

(8) $$P^{-}=R\times P^{+}$$

(9) $$V_{c}=V_{in}\times G=-2jSP^{+}\sin\left(kd\right)\times G$$

With Equations (8) and (9), the total reflected wave, which makes it to be zero at the target frequency, is obtained as below.

(10) $$\bar{P_{r}}=R\times P^{+}+V_{c}\times T=P^{+}\left[R+2SC_{0}\omega \sin\left(\left(\frac{\omega }{c}\right)d\right)\times G\right]$$

(11) $$G_{0}=\frac{-R}{2SC_{0}\omega_{0}\sin\left(\left(\frac{\omega_{0}}{c}\right)d\right)}$$

G is the system gain. G was set to $G_{0}$, which is the value that causes the amplitude of the total reflected signal to be zero at a specific frequency. Assume that the receiving sensors are thin enough that the transmission loss could be neglected.

### 4.3. Plane Array

In this study, a plane array of nine multilayer acoustic absorbers was used. The incident acoustic signal generated by the sound source can be collected by every absorber. Using time-delay separation and collected data from each receiving absorber, the digital controller can calculate the reflected acoustic signal.

(12) $$V_{A\_plane}=(\sum_{i=1}^{i\_max}\sum_{j=1}^{j\_max}V_{A\_ij})/\left(i_{max}+j_{max}\right)$$

(13) $$V_{B\_plane}=(\overset{i\_max}{\underset{i=1}{\sum }}\overset{j\_max}{\underset{j=1}{\sum }}V_{B\_ij})/\left(i_{max}+j_{max}\right)$$

where *i_max* =3 and *j*_*max* = 3. Equations (12) and (13), $V_{A\_plane}$ and $V_{B\_plane}$ are the average values of $V_{A}$ and $V_{B}$ in each single multilayer acoustic absorber. i and j are the component index of the plane array of smart skin absorbers, respectively. $V_{A\_ij}$ and $V_{B\_ij}$ are the measured value of i and j component receiving sensor. Using Equations (12) and (13), incident wave and reflected wave of the plane array of smart skin absorbers are calculated.

(14) $$V_{in\_plane}=V_{B\_plane}e^{-jkd}-V_{A\_plane}=-2jS\left(\omega \right)P_{avr}^{+}\sin\left(kd\right)e^{jkd}$$

(15) $$V_{r\_plane}=V_{A\_plane}e^{-jkd}-V_{B\_plane}=-2jS\left(\omega \right)P_{avr}^{-}\sin\left(kd\right)e^{-2jkd}$$

$V_{in\_plane}$ is the average incident acoustic signal of the plane array and $V_{r\_plane}$ is the average reflected acoustic signal of the plane array. $P_{avr}^{+}$ and $P_{avr}^{-}$ are averages of each amplitude of the incident and reflected acoustic signals, respectively.

As a single absorber is expanded into a plane array, the magnitude of the measured value from each receiving sensor varies, but it is used to determine the direction of the sound source. This study indicated that the sound source was in front of the sensor and that there were no different angles of incidence. This assumption simplifies the control problem. The calculated control value $V_{C\_plane}$ is used as a value for the reflected reduction signal.

(16) $$V_{c\_plane}=V_{in\_plane}\times G=-2jSP_{avr}^{+}\sin\left(kd\right)\times G$$

$V_{c\_plane}$ is the control signal in a plane array of smart absorbers. By replacing the value of $V_{c}$ in Equation (10) with $V_{c\_plane}$, it is possible to obtain a total reflected wave, which makes it to be zero at the target frequency of the flat array type smart skin sensor.

## 5. Experimental Results

**Figure 10:**
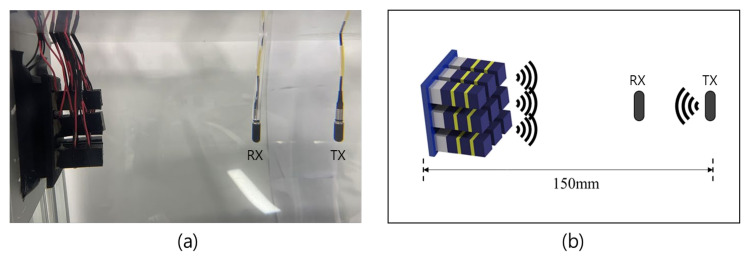
Experimental setting: (**a**) actual setting, (**b**) schematic of the experimental setting.

An experiment with reflected wave reduction control was conducted to evaluate the performance of a plane array with multilayer acoustic absorbers. A reflected reduction experiment is carried out to compare the attenuation rate of active and passive controls. The application of the active control to the plane array with multilayer is allowed for a digital controller to generate a control signal with the same magnitude as the opposite phase, whereas the passive control scheme is entirely dependent on the natural material’s sound-absorbing properties and has no control over them. Experiments on the plane array are set up as shown in Figure 10a. A projector is placed at 150 mm from the front face of the plane array, as shown in Figure 10b.

**Figure 11:**
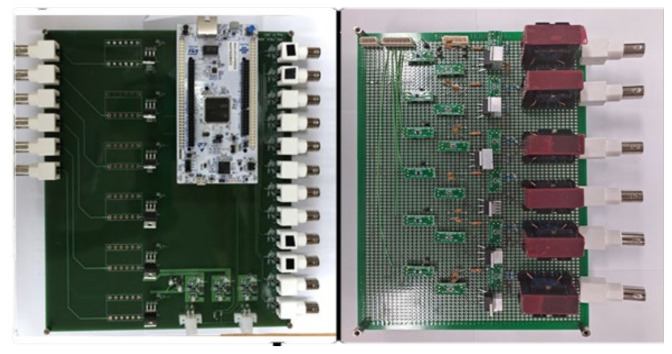
The control circuit of a plane array of smart skin absorbers.

The control circuit (18 receiving channels and 9 transmitting channels) is developed for measuring incident signals and generating an echo control signal, as in Figure 11. The receiver circuit primarily performs signal processing functions, such as filtering and amplifying signals, before transferring them to the controller. The experiments are used to determine the filter’s amplification ratio and cutoff frequency. The control circuit is composed of a DAC (digital-analog converter) element, a transformer and a power op-amp.

**Figure 12:**
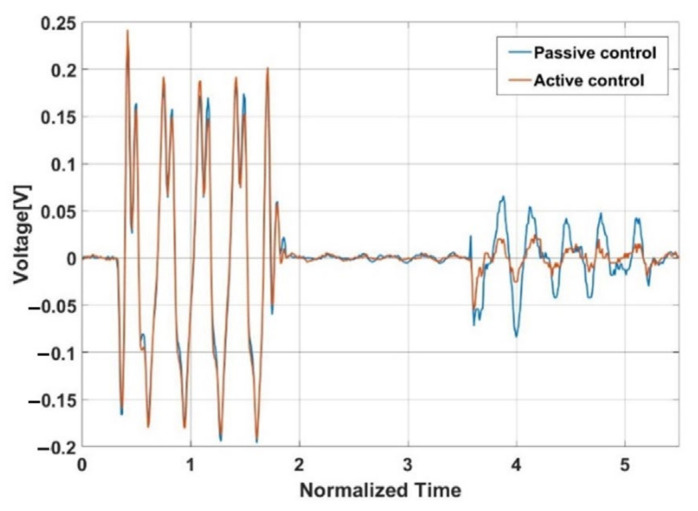
Reflection wave cancellation experiment result.

The performance of a plane array with single absorbers using the active control approach is compared to that of a plane array with no controller (passive control). Figure 12 displays that the source signal is a five-cycle burst sinusoidal signal with 246 mV amplitude, while the amplitude of the reflected acoustic signal with no controller is 74 mV. This means that for a plane array with no active control, the echo reduction is −10.4 dB. A case of applied active control on the same condition presents that the reflected acoustic signal is ~23 mV with a reduction rate of ~−20.58 dB. This means that the proposed plane array with active control shows much higher echo reduction.

**Figure 13:**
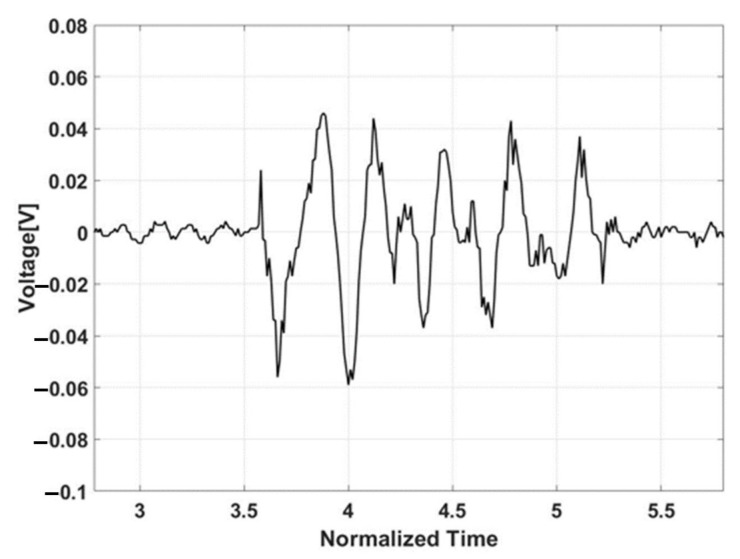
Difference between passive control and active control.

Figure 13 displays the difference in the magnitude of reflected acoustic signal between the passive and active control schemes. It demonstrates that under active control, the measured voltage level in the hydrophone is 30 mV lower on average. Active control performs better in terms of reduction. It means that multilayer acoustic absorbers with a smaller size can respond to low frequency.

## 6. Conclusions

This paper demonstrates reflected wave reduction on the plane array with multilayer acoustic absorbers. Each absorber consists of two receiving sensors and one piezoelectric material. It enables two receiving sensors to collect the time-delayed phase and magnitude of the incident acoustic signal. Expanding the control scheme of a single, smart skin sensor to a plane array is proposed, which allows the system to maintain a suitable echo reduction performance. In a confined environment experiment, a plane array with a digital controller (active control) attenuated reflected acoustic signals for vertically incident acoustic signals. The outcome of the experiment demonstrates a significant point. It is an effective reflected sound reduction of active control. The plane array of multilayer acoustic absorbers has a higher attenuation rate when using active control of ~−10dB.

The purpose of this research is to expand a single, smart skin absorber to a plane array with multilayer absorbers and compare the attenuation rate between passive and active control. Adaptive feedback control of single, smart skin sensors was investigated, as in previous work [14]. It shows attenuation rate performance of ~−36dB. It means that a plane array of smart skin absorbers can also achieve higher attenuation rates using adaptive feedback control. Further, the optimal control of a plane array of smart skin absorbers to achieve a higher attenuation rate will be an interesting research topic.

## Data Availability

Data sharing is not applicable.

## References

[1] Gao N., Lu K. (2020). An underwater metamaterial for broadband acoustic absorption at low frequency. Appl. Acoust..

[2] Jayakumari V.G., Shamsudeen R.K., Rajeswari R., Mukundan T. (2019). Viscoelastic and acoustic characterization of polyurethane-based acoustic absorber panels for underwater applications. J. Appl. Polym. Sci..

[3] Or K.H., Putra A., Selamat M.Z. (2017). Oil palm empty fruit bunch fibres as sustainable acoustic absorber. Appl. Acoust..

[4] Manish R., Fatima S., Tandon N. (2020). An experimental and theoretical study on environment-friendly sound absorber sourced from nettle fibers. J. Build. Eng..

[5] Hoflack M., Van D.B. (2003). Active control of acoustic reflection, absorption, and transmission using thin panel speakers. Noise Vib. Bull..

[6] Wang W., Thomas P.J. (2017). Low-frequency active noise control of an underwater large-scale structure with distributed giant magnetostrictive actuators. Sens. Actuators A Phys..

[7] Kim J., Ji Y., Park Y., Noh E., Ohm W., Choi Y., Kim D., Seo Y. (2017). A study on the low-frequency active echo reduction technology for reducing underwater target echo signal. Korean Soc. Noise Vib. Eng..

[8] Kuo S.M., Morgan D.R. (1999). Active noise control: A tutorial review. Proc. IEEE.

[9] Gentry C.A., Guigou C., Fuller C.R. (1997). Smart foam for applications in passive-active noise radiation control. J. Acoust. Soc. Am..

[10] Cong C., Tao J., Qiu X. (2018). A multi-tone sound absorber based on an array of shunted loudspeakers. Appl. Sci..

[11] Lee J., Kim J., Rhee C., Jo C., Choi S. (2002). Noise reduction of passive and active hybrid panels. Smart Mater. Struct..

[12] DeAngelis D., Schulze G. (2015). Performance of PIN-PMN-PT single crystal piezoelectric versus PZT8 piezoceramic materials in ultrasonic transducers. Phys. Procedia.

[13] Wu Z., Bao X., Varadan K.V., Varadan V.V. (1993). Broadband active acoustic absorbing coating with an adaptive digital controller. Smart Mater. Struct..

[14] Lee H., Park H., Park K., Yi H. (2020). Application of adaptive wave cancellation underwater to a piezoelectric-material-based multilayer sensor. Sensors.

[15] Owsley N.L., Swope G.R. (1981). Time delay estimation in a sensor array. IEEE Trans. Acoust..

[16] Carter G.C. (1981). Time delay estimation for passive sonar signal processing. IEEE Trans. Acoust..

[17] Quazi A.H. (1981). An overview on the time delay estimate in active and passive systems for target localization. IEEE Trans. Acoust..

[18] Lee J., Lee C.H., Bae J., Paeng D.G., Choe M.H., Kim W.H. (2013). Parametric array signal generating system using transducer array. J. Acoust. Soc. Korea.

[19] Lee O., Son Y., Kim K. (2002). Performance enhancement of underwater acoustic communication system using hydrophone transmit array. J. Acoust. Soc. Korea.

[20] Noh E., Chun W., Ohm W.S., Been K., Moon W., Chang W., Yoon H. (2017). Iteration-based array analysis for conceptual design of active sonar arrays. Trans. Korean Soc. Noise Vib. Eng..

